# A Comparative Study of the Typing Performance of Two Mid-Air Text Input Methods in Virtual Environments

**DOI:** 10.3390/s23156988

**Published:** 2023-08-06

**Authors:** Yueyang Wang, Yahui Wang, Xiaoqiong Li, Chengyi Zhao, Ning Ma, Zixuan Guo

**Affiliations:** 1 The School of Medical Technology, Beijing Institute of Technology, Beijing 100081, China; yueyang_wang@outlook.com; 2 The School of Life Science, Beijing Institute of Technology, Beijing 100081, China; aeople@126.com; 3 Faculty of Psychology, Beijing Normal University, Beijing 100875, China; 202128061068@mail.bnu.edu.cn (C.Z.); 202228061002@mail.bnu.edu.cn (Z.G.); 4 Department of Design, Kyiv National University of Technologies and Design, 01011 Kyiv, Ukraine; maningyhh@163.com

**Keywords:** usability testing, text input, virtual reality, pointing devices, keyboards, gestural input

## Abstract

Inputting text is a prevalent requirement among various virtual reality (VR) applications, including VR-based remote collaboration. In order to eliminate the need for complex rules and handheld devices for typing within virtual environments, researchers have proposed two mid-air input methods—the trace and tap methods. However, the specific impact of these input methods on performance in VR remains unknown. In this study, typing tasks were used to compare the performance, subjective report, and cognitive load of two mid-air input methods in VR. While the trace input method was more efficient and novel, it also entailed greater frustration and cognitive workload. Fortunately, the levels of frustration and cognitive load associated with the trace input method could be reduced to the same level as those of the tap input method via familiarity with VR. These findings could aid the design of virtual input methods, particularly for VR applications with varying text input demands.

## 1. Introduction

Virtual reality (VR) has become a popular tool for entertainment in gaming and for enhancing office productivity, collaboration, applications, and training, as well as in the training and interaction of special robots [1,2,3,4,5,6,7,8,9]. Text input is an essential element of communication, recording, and reporting in VR.

### 1.1. Text Input in VR

Recent studies have addressed the challenges of text input in the context of VR. VR input technologies primarily rely on auxiliary devices such as physical keyboards, touch screens, controllers, and data gloves, each offering unique advantages and disadvantages.

Physical keyboards enable high-speed typing, but their lack of portability is a significant drawback [10]. The input speed of physical keyboards could reach 30 words per minute (WPM), with an accuracy rate of around 80% [11]. Touch screens and controllers offer better portability and typing speeds exceeding 8 WPM, but they are heavy and can detract from the user’s sense of immersion in the VR environment [12,13,14,15,16,17,18,19,20,21]. Additionally, some text input methods based on controllers involve a substantial learning cost. Users need to memorize the actions corresponding to each letter to effectively use the controller for input [22]. Some researchers have proposed a pen-based virtual reality text input method that does not require spatial positioning and allows for direct actions such as rotating the pen, achieving a maximum input speed of 6.6 WPM. Despite presenting a novel text input model, this study fell short in terms of portability and input speed [23]. Data gloves are more portable and allow users to enter letters using glove sensors based on specific input rules, but the learning curve is steep [24,25,26,27,28]. Image-recognition-based handwriting input is a new method of text entry. Its principle involves using neural networks to recognize gesture trajectory images, with users needing to write the desired input in mid-air. Currently, the recognition accuracy of this technology can reach up to 87.1% [29]. Regrettably, no study has accurately reported the input speed for this method to date. Another solution involves handwriting recognition based on flexible pressure sensors, where neural networks are used to recognize writing trajectories on flexible materials for input, achieving speeds of up to 12.3 WPM [30]. However, in previous studies, compared to typing, handwriting input often fell behind in terms of input speed and user experience [31,32]. Additionally, there are VR input methods based on head or eye motion [17], but these can cause motion sickness [33,34,35,36]. Voice-based input is also one of the virtual reality text input methods that has received attention in recent years. The speed of voice input is fast, with a WPM of 60–70. However, voice input is greatly influenced by the sound environment. When the ambient noise reaches 70 decibels, only 37.5% of users continue to use voice input [37]. Therefore, we believe that a suitable VR input method should strike a balance between typing efficiency, portability, and motion sickness. The text input solutions in VR mentioned in the above studies have each shown their respective drawbacks in terms of input efficiency, portability, and user experience. The mid-air text input methods proposed in this study are freehand text input methods. Our research focused on this interaction method, demonstrating the feasibility of mid-air text input methods in VR.

### 1.2. VR Text Input via Mid-Air Interaction

Mid-air text input methods offer distinctive advantages in the realm of VR. Mid-air interaction has evolved to include freehand interactive methods with hand-tracking sensors like Leap Motion, which allow users to express themselves with their hands. Studies have demonstrated that this natural hand interaction greatly enhances the immersion and engagement experience in VR [38,39,40].

Researchers have developed input techniques for large displays using mid-air interaction, such as wall displays or televisions [41,42,43,44,45]. Various researchers have determined that this input technique holds potential in VR. For instance, a tap VR input method was evaluated that utilized “direct interaction” with no space between the keyboard and fingertips, and participants achieved a typing speed of 14 WPM [46]. A recent study also mentioned the trace input method for text input in virtual reality and conducted a preliminary evaluation, suggesting that the race input method holds promise. However, this study did not objectively compare the trace input method with other traditional input methods, so the advantages and disadvantages of the two different input methods in VR are still unknown [47]. Although mid-air interaction is a promising technology for VR immersion, it may also cause fatigue. Even with limited mid-air interaction, participants may suffer from discomfort, known as “gorilla arm syndrome”, which is the fatigue resulting from holding one’s hands up for extended periods [48]. Our research objectively compared the trace input method and the tap input method in terms of performance, experience, and workload among different VR user groups, providing evidence to understand the strengths and weaknesses of the two input methods and their suitable user groups.

### 1.3. Typical Input Methods: Tap and Trace

The tap input method (Tap) and the trace input method (Trace) are two prominent text input mechanisms employed in physical user interfaces [32,49]. With Tap, users input each letter by tapping on the keyboard individually. On the other hand, Trace involves the participant sliding their finger over all the letters of a word on the keyboard to input the whole word. Compared to Tap, Trace exhibits superior performance on various devices such as smartphones, smartwatches, and augmented reality [31,32,36,50,51]. These studies have established that Trace outperforms Tap in numerous interactive settings, including touch screens, head-based virtual screens, and controllers. However, the prior applications of Tap and Trace did not encompass mid-air interaction scenarios in which text input necessitated “indirect interaction” (users could not directly touch the front virtual keyboard).

Mid-air Tap and Trace are freehand interactive approaches that require users to raise their arms and move their hands to interact indirectly with a virtual keyboard. When utilized in VR, mid-air input methods necessitate users to control their upper limbs more precisely, which could prove challenging and exhausting. Although indirect interaction can reduce the accuracy of mid-air interaction, it also increases the diversity of interactive positions [52,53,54]. Hence, the efficiency of these VR mid-air input methods remains largely unexplored. This study postulated that the mid-air Trace method would outperform mid-air Tap in VR. We anticipated that the trace method would exhibit a higher input efficiency, since drawing a trace is a quicker interaction than tapping each button sequentially. Additionally, fatigue would be minimized with Trace, because the interaction time for inputting the same text would be reduced.

The experience of users with VR may also influence the performance of these input methods. A recent study concluded that more extensive experience with large displays resulted in fewer errors [55]. Firstly, we posited that VR-experienced users may perform better in mid-air input tasks, since they are more acclimatized to interacting with objects in VR. Secondly, as VR familiarity grows, participants may have higher expectations for the efficiency of text input in VR. A user who is well-versed in VR may prefer the trace input method due to its efficiency. Hence, this study compared two mid-air text input methods in VR, focusing on performance, subjective feedback, and cognitive load, among two groups of participants who differed in their prior VR experience. With the emergence of metaverse platforms, users can engage in social interactions via immersive experiences in the virtual world, breaking down social barriers. In the virtual world, everyone can communicate through natural interactions via gesture interaction and feel a sense of equality and freedom in the metaverse. This study lays the foundation for researching face-to-face immersive input experiences for users, which holds strong potential for establishing social relationships based on the metaverse.

## 2. Materials and Methods

### 2.1. Participants

After obtaining approval from the Ethics Committee of Tsinghua University, the study recruited a total of thirty-eight right-handed participants. However, due to incomplete recording, only the data of 35 valid participants (17 males and 18 females) aged between 18 and 29 years (*M* = 21.43, *SD* = 3.211) were included in the final analysis. Participant selection was based on a standard protocol. All participants possessed a bachelor’s degree or above or were currently studying in a university. Participants were categorized into two groups based on their previous experience with virtual reality devices. The experienced group included individuals who had used virtual reality devices five times or more with a total duration of more than one hour over the past year. Alternatively, participants who reported no experience with virtual reality devices were recruited into the inexperienced group. None reported prior experience using Trace, while all participants reported a high frequency of using the QWERTY keyboard (similar to that used to implement Tap) on smartphones in the past. Furthermore, none of the participants claimed any experience with text entry in virtual reality or expertise with Leap Motion. The inexperienced group comprised 19 participants (10 females and 9 males), while the experienced group included 16 participants (8 females and 8 males). Finally, all participants had normal or corrected vision and were free from physical diseases. Participation in the study was rewarded with RMB 150.

### 2.2. Apparatus

In this study, we constructed a virtual reality system that consisted of an advanced VR headset (HTC Vive Pro Eye; 110° horizontal field of view; 1440 × 1600 pixels per eye; 90 Hz), combined with an eye-tracker (110° trackable field of view; 120 Hz) and a computer (CPU: i7-10875 H 2.30 GHz, GPU: GTX 2070 SUPER).

**Figure 1:**
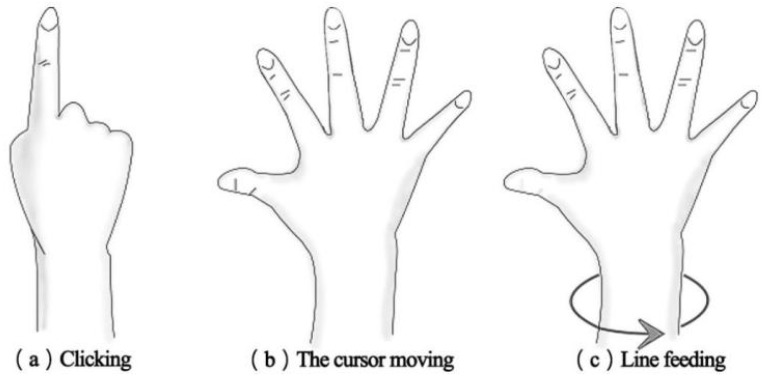
Three gestures.

In order to enable gesture recognition, we developed a custom program using Leap Motion (140° × 120° field of view; 10–80 cm interaction depth), which we positioned on the VR headset. We defined three distinct gestures to control the cursor movement, including clicking letters, cursor movement, and line feeding, as illustrated in Figure 1.

**Figure 2:**
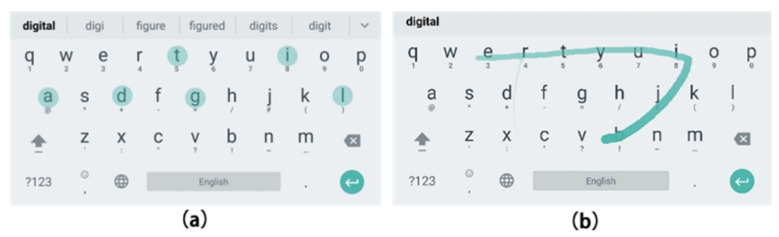
The tap input method (**a**) and the trace input method (**b**).

We utilized an Android 5.5.1 virtual machine operating the QWERTY virtual keyboard, namely the Google Input Application, which supported both Trace and Tap input methods. As shown in Figure 2, Tap involved individually tapping each letter in the given word prompt, such as the word “digital”, with seven letters. Participants moved the cursor via gesture (b) and tapped each letter one by one using gesture (a). In contrast, with Trace, participants drew a continuous trace across all letters of the word prompt in order. To begin, participants placed the cursor on the initial letter of the word using gesture (b) and clicked the first letter with gesture (a). Subsequently, they sustained the clicking and moved the cursor across all letters in turn, with a trace appearing on the path of the cursor. The trace virtual keyboard then processed the trace in real time, displaying the predicted words on the virtual keyboard. Participants could then move the cursor and select their desired word from the options bar using gestures (a) and (b). If the correct word prediction was the first option, participants could confirm and switch to the next line using gesture (c), which involved turning their palm 180° and back.

**Figure 3:**
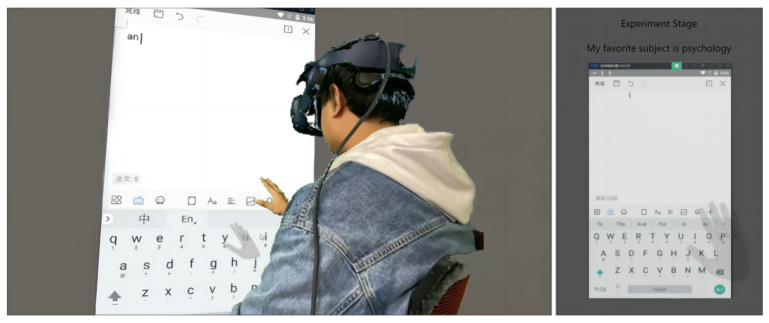
Virtual environment.

The participants were able to observe the real-time user interface (UI) of the virtual machine through a head-mounted display (HMD), with phrases being sequentially displayed on top of it. Whenever a phrase was completed by the participants, they would then press the trigger button on the controller to move on to the next phrase. The experimental setting adopted a gray background, intended to facilitate participants’ concentration. The participants’ hands were displayed via a model with 50% transparency, as depicted in Figure 3.

**Figure 4:**
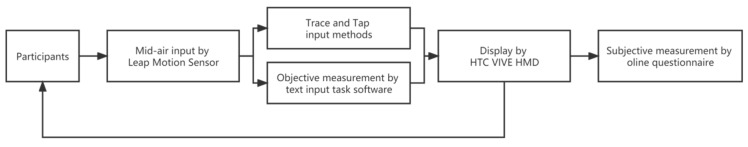
Experimental system schematic representation.

Fifteen practice phrases and ten experimental phrases were randomly selected for each method and for each participant, taken from MacKenzie and Soukoreff’s phrase list [56]. For each method, each user had different phrases. These phrases contained only 16 to 43 lowercase letters. A schematic representation of the experimental system used in this study is depicted in Figure 4.

### 2.3. Procedure

Upon providing informed consent, the participants devoted a minimum of 30 min to acquainting themselves with the proper utilization of the tap and trace input techniques via Leap Motion. This practice persisted until the completion of five precise phrases entered using each method. Such measures ensured that the eventual experimental data exclusively reflected variances between the input methods, as opposed to unfamiliarity with the Leap Motion system. Following this, participants received concise instructions on employing the VR headset and controllers.

The first step entailed the participants entering practice phrases using one of the input methods for approximately 20 min, amounting to a total of 15 phrases. The second step involved a rest period to counteract any potential impact of fatigue. Continuing to the third step in the experimental phase, participants endeavored to input 10 phrases with celerity and precision within an approximate 10 min timeframe, using the input approach with which they had previously familiarized themselves during the first step. Upon accomplishing the aforementioned tasks, the participants removed the HMD and dedicated around 10 min to filling out questionnaires. After this, in order to alleviate possible fatigue, they underwent another rest phase before repeating the entire experiment by performing the same steps as outlined above, except with the alternative input technique. Demographic data, such as age and gender, were obtained following the completion of the abovementioned stages. Each break lasted for 15 min; however, participants were at liberty to request additional rest time. Consequently, seven participants opted for longer break intervals, ranging from 18 to 23 min. Each person expended around 180 min performing the experiment, and input errors were not allowed to be rectified. To promote the ease of eventual data analysis, the participants switched to the subsequent row upon concluding each word, instead of each phrase. Table 1 shows the description of the experimental stages.

### 2.4. Design

The present study employed a 2 × 2 mixed design, with the input method (Trace vs. Tap) serving as the within-subject factor, while VR experience (experienced group vs. inexperienced group) was employed as the between-subject factor. The dependent variables for this study were typing performance, subjective experience, and workload.

#### 2.4.1. Typing Performance

To assess the typing performance, we measured typing speed (WPM), typing accuracy (WAcc), and typing efficiency. The WPM formula was

WPM = (|*T*| − 1)/*S* × 12 (1)

where *T* denotes the number of characters that were input, while *S* refers to the duration of the input (measured in seconds) [57]. 

WAcc was determined by calculating the percentage of correctly entered words (*C*) from the total number of words (*T*) [58]. The WAcc formula was

(2) WAcc=C/T

However, the efficiency of an input method could not be comprehensively assessed by merely measuring the speed or accuracy independently. Rather, it was necessary to consider the trade-off between these two factors. Previous studies permitted participants to correct input errors, so if all errors could be amended, the speed would indicate the effectiveness of the method. However, practical experiments showed that errors were still committed by participants, prompting the need to take the trade-off into account [31,32]. Fitts’ law defines the speed–accuracy trade-off as the quotient of the task’s degree of difficulty divided by the task’s mean time, which measures the number of bits of task difficulty that an interface can process per second [31,32]. Similarly, in this study, we examined the typing efficiency (TE) by calculating the product of the input speed (without error correction) and the input accuracy, thus indexing how many words the user could accurately enter with the specific input method per unit time. The TE formula was

(3) TE=WPM×WAcc

In this study, we performed repeatability tests by comparing the typing accuracy during the practice phase and the experimental phase, aiming to assess the impact of the learning effect on the input performance. When participants were assigned to one of the input methods, they first entered 15 sentences during the practice phase. After a rest period, they continued to input 10 sentences during the experimental phase. We then compared the input accuracy of this method between the practice and experimental stages to perform the repeatability test. The same procedure was applied when participants used the other input method. We ensured that all sentences used in the experiment were selected randomly. Also, the order of the input methods was balanced using a Latin square design.

#### 2.4.2. Subjective Experience

We evaluated the users’ subjective experiences by measuring their user experience, susceptibility to motion sickness, and perceived exertion.

To evaluate the participants’ user experiences for each method, the User Experience Questionnaire (UEQ) was implemented. This instrument comprises 26 pairs of contrasting attributes, spread across six facets—attractiveness (referring to the overall appeal of the product); perspicuity (pertaining to the level of difficulty of use); dependability (encompassing factors such as predictability, controllability, and security); stimulation (measuring the level of excitement induced by interaction with the product); novelty (assessing the creativity of the product design); and efficiency (evaluating the effectiveness of interaction efforts). Participants responded using a scale with seven options, ranging from negative to positive. Higher scores indicated a better user experience [59].

To assess motion sickness caused by VR, the VR sickness questionnaire (VRSQ) was employed. This nine-item scale evaluates two facets—oculomotor and disorientation factors—through four response options. Based on the questionnaire responses, a motion sickness score ranging from 0 to 100 was obtained, with higher scores indicating more severe motion sickness [60].

The Borg CR10 scale was utilized to gauge the participants’ overall perceived physical exertion. This single-item scale has a range of 0 to 10, with higher scores indicating a higher perceived level of exertion [61].

#### 2.4.3. Workload

We assessed the workload through subjective and cognitive means. NASA TLX and pupil diameter were employed to measure these variables [62,63].

NASA TLX is a scale for evaluating the subjective workload associated with various tasks. Participants are rated on factors such as mental and physical demands. A higher score indicates a heavier workload [62]. In our study, we employed this tool to evaluate and compare the scores of two different virtual reality (VR) input methods across these workload dimensions. This included an assessment of the overall workload score as well as the scores for each individual dimension. We conducted a variance analysis to identify significant differences between these two input methods across various workload dimensions.

In addition, the mean pupil diameter is an essential indicator for estimating cognitive load during input tasks. This study collected data on the right-eye pupil diameter of the participants while performing text input tasks at a sampling rate of 90 Hz. We calculated the mean pupil diameters individually during the text input tasks completed with different input methods. The pupil diameter is an indicator of cognitive load level, with larger diameters corresponding to higher cognitive loads [63].

## 3. Results

In this study, the learning effect was assessed using a paired T-test, while other dependent variables were analyzed through a 2 × 2 mixed-design ANOVA. The NASA TLX was also analyzed using separate ANOVA tests for each dimension. For the overall rating, a weighted approach was applied, whereby the product of each subscale score and the workload factor paired-choice task’s weight score was divided by the sum of the weights. The effect size was calculated using partial eta squared (*η_p_*^2^) in the ANOVA tests. A follow-up analysis was carried out through simple main effects to explore significant interactions.

### 3.1. Typing Performance

#### 3.1.1. Words Per Minute (WPM)

**Figure 5:**
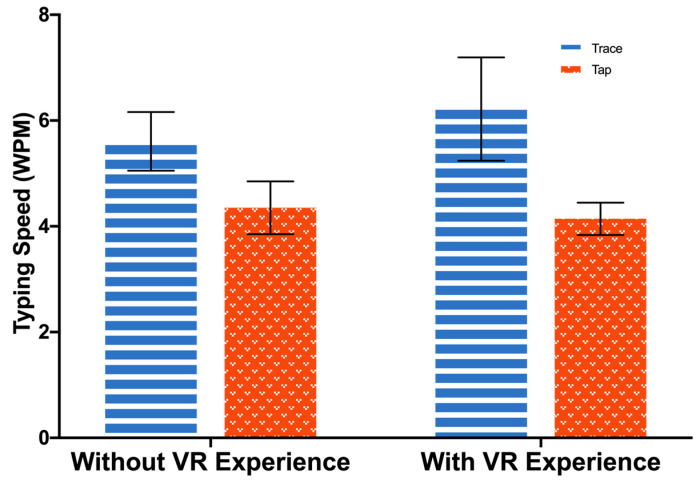
Words per minute. Error bars represent 95% confidence interval (CI).

Text entry methods had a significant main effect on the WPM. Participants typed faster using Trace (*M* = 5.9, *SD* = 1.5) than Tap (*M* = 4.3, *SD* = 0.9): *F*(1, 33) = 68.4, *p* < 0.001, *η_p_*^2^ = 0.67. There was no significant difference between the experienced group and the inexperienced group in terms of typing speed: *F*(1, 33) = 0.3, *p* = 0.58, *η_p_*^2^ = 0.01. A significant interaction between the text entry method and VR experience was found: *F*(1, 33) = 4.1, *p* = 0.05, *η_p_*^2^ = 0.11. The follow-up analysis revealed that Trace produced a higher typing speed than Tap in both groups, *p* < 0.001. Figure 5 shows the typing speed for each set of experimental conditions.

#### 3.1.2. Word Accuracy

The text entry method had a significant main effect on the WAcc. Participants input text more accurately with Tap (*M* = 0.9, *SD* = 0.1) than Trace (*M* = 0.8, *SD* = 0.1): *F*(1, 33) = 18.3, *p* < 0.001, *η_p_*^2^ = 0.36. There was no significant difference between the experienced group and the inexperienced group in terms of typing accuracy: *F*(1, 33) = 0.3, *p* = 0.57, *η_p_*^2^ = 0.01. Neither was there an interaction effect between the input method and the VR experience: *F*(1, 33) = 0.0, *p* = 0.99, *η_p_*^2^ < 0.001.

#### 3.1.3. Typing Efficiency

The text entry method had a significant main effect on the typing efficiency. Participants input text more efficiently with Trace (*M* = 4.4, *SD* = 1.3) than Tap (*M* = 3.7, *SD* = 1.1): *F*(1, 33) = 13.7, *p* = 0.001, *η_p_*^2^ = 0.29. There was no significant difference between the experienced group and the inexperienced group in terms of typing efficiency: *F*(1, 33) = 0.01, *p* = 0.94, *η_p_*^2^ < 0.001. Neither was there an interaction effect between the input method and the VR experience: *F*(1, 33) = 2.0, *p* = 0.17, *η_p_*^2^ = 0.06.

#### 3.1.4. Effect of Learning on Typing Accuracy

The WAcc was compared by a paired *t*-test between the practice and experimental tasks for both input methods. There were no effects of learning on the typing accuracy, *p* > 0.42.

### 3.2. Subjective Experience

#### 3.2.1. User Experience

The text entry method had two significant effects on user experience. Participants reported a higher perspicuity score when using Tap (*M* = 2.2, *SD* = 1.0) compared to Trace (*M* = 1.6, *SD* = 1.2): *F*(1, 33) = 7.1, *p* = 0.01, *η_p_*^2^ = 0.18. Participants also reported a higher novelty score when using Trace (*M* = 2.2, *SD* = 0.8) compared to Tap (*M* = 1.5, *SD* = 1.2): *F*(1, 33) = 15.6, *p* < 0.001, *η_p_*^2^ = 0.32. The text entry method had no significant effect on other facets, *p* > 0.13. There was no significant difference between the experienced group and the inexperienced group in terms of the six facets of user experience, *p* > 0.28. No interaction effects were found, *p* > 0.25.

#### 3.2.2. Motion Sickness

There was no significant difference in terms of motion sickness between each method: *F*(1, 33) = 0.02, *p* = 0.88, *η_p_*^2^ = 0.001 and *F*(1, 33) = 0.2, *p* = 0.66, *η_p_*^2^ = 0.01, respectively. Additionally there were no significant interaction effects: *F*(1, 33) = 1.8, *p* = 0.19, *η_p_*^2^ = 0.05.

A further analysis of the VRSQ’s sub-dimensions (oculomotor and disorientation factors) found no significant effects of the input method on the VR experience, *p* > 0.65. No significant interaction effect was found, *p* > 0.11.

#### 3.2.3. Perceived Exertion

No significant main effect on perceived exertion was found for the methods or VR experience: *F*(1, 33) = 0.8, *p* = 0.39, *η_p_*^2^ = 0.02 and *F*(1, 33) = 0.7, *p* = 0.40, *η_p_*^2^ = 0.02, respectively. There was no significant interaction effect: *F*(1, 33) = 0.06, *p* = 0.81, *η_p_*^2^ = 0.002.

### 3.3. Workload

#### 3.3.1. Subjective Workload

**Figure 6:**
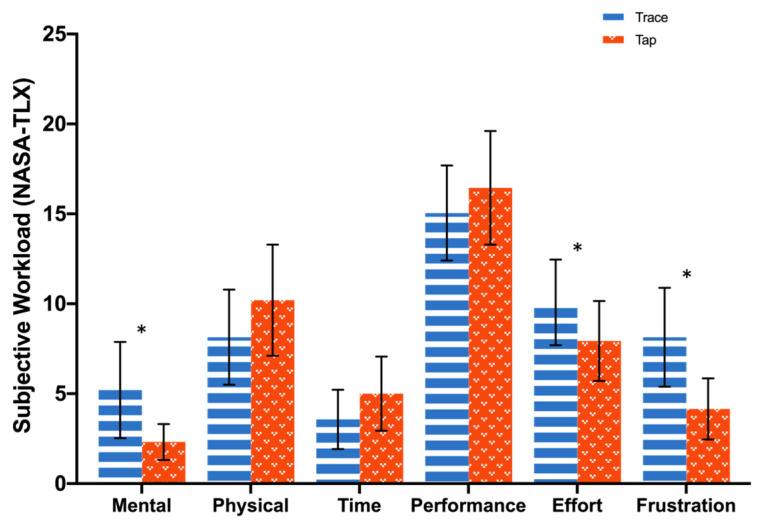
Subjective workload (according to the six facets of the NASA-TLX scale) of participants when using different text entry methods. Error bars represent 95% CI. The symbol * indicates a statistically significant main effect.

The text entry method had a significant main effect on the overall task workload: *F*(1, 33) = 4.2, *p* = 0.05, *η_p_*^2^ = 0.11 (Trace: *M* = 42.7, *SD* = 15.2; Tap: *M* = 36.0, *SD* = 15.1). The input method had three significant main effects in terms of effort, mental load, and frustration. The effort score with Trace (*M* = 10.1, *SD* = 6.9) was higher than with Tap (*M* = 7.9, *SD* = 6.5): *F*(1, 33) = 5.3, *p* = 0.03, *η_p_*^2^ = 0.14. The mental load reported by participants with Trace (*M* = 5.2, *SD* = 7.8) was higher than with Tap (*M* = 2.3, *SD* = 2.9): *F*(1, 33) = 5.6, *p* = 0.02, *η_p_*^2^ = 0.15. The frustration reported by participants when using Trace (*M* = 8.1, *SD* = 8.0) was higher than when using Tap (*M* = 4.2, *SD* = 5.0): *F*(1, 33) = 6.3, *p* = 0.02, *η_p_*^2^ = 0.16. A significant interaction in terms of frustration between the method and the VR experience was found: *F*(1, 33) = 5.3, *p* = 0.03, *η_p_*^2^ = 0.14. No significant main effect between the experienced group and the inexperienced group was found, *p* > 0.18. The follow-up analysis revealed that participants without VR experience reported higher levels of frustration when using Trace than when using Tap, *p* = 0.01. The participants with VR experience reported lower levels of frustration when using Trace than when using Tap, *p* = 0.04. Figure 6 shows the statistical analysis of the subjective workload. Figure 6 does not distinguish between participants but instead presents the average scores and standard deviations for all test participants.

#### 3.3.2. Cognitive Workload (Mean Pupil Diameter)

**Figure 7:**
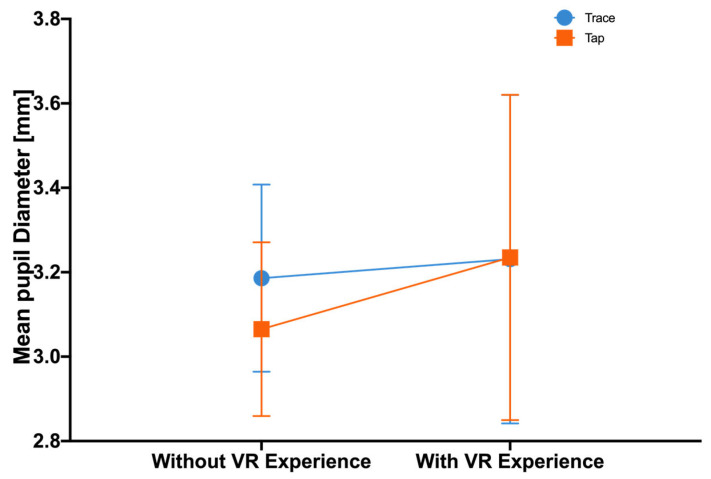
Mean pupil diameter. Error bars represent 95% CI.

Pupil diameter is considered a significant indicator of cognitive load during typing tasks. The text entry method had a significant effect on pupil diameter, with Trace (*M* = 3.2, *SD* = 0.1) presenting higher values than Tap (*M* = 3.2, *SD* = 0.1): *F*(1, 33) = 7.3, *p* = 0.01, *η_p_*^2^ = 0.18. No significant main effect between the experienced group and the inexperienced group on pupil diameter was detected: *F*(1, 33) = 0.3, *p* = 0.59, *η_p_*^2^ = 0.01. A significant interaction was found between the input method and the VR experience: *F*(1, 33) = 8.2, *p* = 0.007, *η_p_*^2^ = 0.20. The follow-up analysis revealed that participants without VR experience had a larger mean pupil diameter when using Trace (*M* = 3.2, *SD* = 0.1) compared to Tap (*M* = 3.1, *SD* = 0.1), *p* < 0.001. Figure 7 shows the mean pupil diameter.

## 4. Discussion

Based on the outcomes of TE, user experience, and workload, both input methods exhibited benefits and drawbacks in various areas. Overall, the results indicated that the text entry methods had significant main effects. Additionally, in terms of certain indicators such as WPM, frustration, and cognitive workload, we observed notable interactions between the input method and the virtual reality experience.

### 4.1. The Performance of the Input Methods

Through prolonged practice, satisfactory results in terms of both speed and accuracy can be achieved through physical keyboard and touch interfaces in physical interactions. However, in VR, there exists no physical medium for tapping and tracing. With Tap in VR, the user input each individual letter by tapping on virtual keys, resulting in a higher accuracy at the expense of input speed. In contrast, Trace required the user to input a word by drawing with a trace through the relevant letters, which led to faster typing at the cost of reduced accuracy. As expected, Trace outperformed Tap in terms of speed, whilst Tap exceled in accuracy. In this study, we utilized typing efficiency (TE) to analyze the speed–accuracy trade-off and provide a comprehensive assessment of the true performance of these input methods. After a thorough evaluation, we concluded that Trace was more effective than Tap in promoting typing efficiency.

A meaningful interaction between variables indicated that the VR experience had a larger impact on the input speed of Trace compared to Tap. Figure 5 illustrates the disparity between the VR-experienced and -inexperienced participants in terms of their input speed. The VR-experienced participants were able to input text more swiftly when using Trace compared to VR novices, while no significant difference was observed when using Tap. This could be attributed to the proficiency that the experienced users possessed in mapping physical hand movements to their virtual reality counterparts, enabling them to manipulate virtual objects with greater ease and finesse. As Trace necessitated drawing a continuous trace to input words, it required more precise control than Tap, which may have also contributed to the observed differences in input speed.

According to the literature, both the trace and tap input methods exhibit faster input speeds for smartwatches compared to smartphones [31,32]. In contrast to current research, we discovered that smartphones had faster input speeds than VR mid-air input. It appears that as the size of a device’s user interface increases, the input speed decreases. Inputting on smartwatches only requires finger movement, while inputting on smartphones requires more finger dexterity, and mid-air input as a type of gestural input method in VR demands upper-limb movement. The range of movement of typing may be a crucial factor affecting input speed. The literature also suggests that on smartphones, Trace is more precise than Tap [31], whereas the opposite holds true on smartwatches [49]. In this study, Tap was more precise than Trace. Drawing a trace in one attempt may be difficult on small user interfaces (UIs) such as those on smartwatches, and Trace, as a type of mid-air input method, may cause inevitable limb shaking, ultimately leading to mistouching issues. We believe that Trace UIs require redesigns for use with small smart devices and mid-air input. Optimizing button size and spacing, highlighting the button to be selected, and using a fuzzy word recognition algorithm to improve fault tolerance in virtual mid-air keyboards may be plausible solutions.

### 4.2. The Subjective Experience of the Input Methods

The Trace input method offers a fresh and inventive experience for users, with its distinctive interactive features attracting many willing participants. Conversely, Tap’s input method is characterized by its clarity, making it particularly accessible to beginners. Although neither method is known to induce motion sickness, this study found that continuous mid-air input tasks lasting 10 min elicited a perception of severe exertion. Therefore, VR applications utilizing mid-air input methods should limit the duration and frequency of their use. Subsequent research could involve designing an input task that explores the performance of these input methods under lower-frequency and -duration conditions.

### 4.3. The Workload of the Input Methods

In Figure 6, it was demonstrated that VR-proficient users reported experiencing lower levels of frustration than VR novices when using Trace; however, there was little variation observed between the two groups when using Tap. Drawing continuous traces in VR proved to be more manageable for experienced VR users, while tapping presented no obstacle for either group. As depicted in Figure 7, the experienced VR users’ average pupil diameter was larger than that of the novices when using Tap, but the diameters were more or less the same for Trace. While Tap may have been more user-friendly for VR beginners, it failed to meet the high text input performance expectations of the experienced users. Th experienced participants expressed a strong desire to complete text input tasks efficiently, but the slow speed of Tap increased their anxiety and required more effort and willpower, ultimately resulting in a higher cognitive workload. The results implied that while Trace may have imposed extra cognitive effort and irritation on VR novices, these adverse effects gradually diminished with greater exposure to VR technology, thereby transforming Trace into a superior VR input method.

## 5. Conclusions and Future Work

### 5.1. Conclusions

This research presented a comparative analysis of two freehand mid-air input techniques in VR among both novice and experienced participants. Despite the high portability and minimal motion sickness associated with both gestural input methods, their typing efficiency proved to be rather low. The trace method outperformed the tap method in terms of speed, but no significant differences were observed concerning the fatigue caused by both techniques. This research introduced a new parameter for assessing performance, namely TE, which comprehensively accounted for the speed–accuracy trade-off, and determined that Trace was more effective than Tap. In the future, the TE may be considered as another index for evaluating the performance of input methods, as some methods may have different advantages in terms of speed or accuracy. Contrary to expectations, VR experience only affected the workload, but not the performance or subjective experience. This study lays the groundwork for mid-air text input research in VR with the following conclusions:

(1) Regardless of VR experience, participants displayed greater efficiency in text input when employing the trace input technique. The trace method was deemed more novel by users, while the tap method was more easily comprehensible.
(2) Neither of the VR input techniques induced significant motion sickness, yet both were perceived to require considerable exertion. It is advised to employ short texts for mid-air input tasks in VR.
(3) All participants reported a heightened level of subjective workload while using the trace input method; however, after gaining some experience with VR, their level of frustration decreased to match that of the tap input method.
(4) Participants who lacked experience with VR exhibited lower cognitive workloads when using the tap input method; however, after gaining some experience with VR, their cognitive workloads increased to match those required by the trace input method.

### 5.2. Limitation

There were discrepancies in the English proficiency levels between the participants. Additionally, the participant sample seemed to have a lack of diversity. Initially, all participants were separated into two groups based on their VR experience rather than their English proficiency. The Trace task necessitated that participants draw a continuous line, which required familiarity with the spelling of the words they input. Participants with a limited grasp of English may have encountered more errors while completing the Trace task. Secondly, the study only recruited college students or individuals with a bachelor’s degree or higher, and so did not accurately represent other demographic groups. This inadequacy reduced the ecological validity of the study to some degree. Lastly, the participants were more familiar with the Tap task than the Trace task, which may have resulted in the underestimation of the potential of Trace. To address this issue, we may consider asking invited participants to practice using Trace on their smartphones for 30 min every day for a week leading up to the trial to ensure their familiarity with it.

## Figures and Tables

**Table 1:** Description of the experimental stages.

No.	Stage	Duration (min)	Description
1	Pre-experiment practice	30	Familiarization with tap and trace input methods, entering five precise phrases using each method with Leap Motion
2	VR equipment training	As needed	Instruction on employing the VR headset and controllers
3	Practice before experiment	20	Entering 15 phrases using one of the input methods for familiarization
4	Rest	15	Rest period to counteract any potential impact of fatigue
5	Experiment	10	Inputing 10 phrases with precision and celerity using the input method practiced before
6	Filling out questionnaires	10	Removing the HMD and completing the questionnaires
7	Rest	15 (or longer)	Rest period to alleviate possible fatigue
8	Second round of experiment	40	Same as steps 3–6, but with the alternate input method
9	Collection of demographic data	As needed	Collection of data such as age and gender
